# Application of Microfluidics for Bacterial Identification

**DOI:** 10.3390/ph15121531

**Published:** 2022-12-09

**Authors:** Fraser Daniel, Delaney Kesterson, Kevin Lei, Catherine Hord, Aarti Patel, Anastasia Kaffenes, Harrshavasan Congivaram, Shaurya Prakash

**Affiliations:** 1 Department of Mechanical and Aerospace Engineering, College of Engineering, The Ohio State University, Columbus, OH 43210, USA; 2 Center for Life Sciences Education, The Ohio State University, Columbus, OH 43210, USA; 3 Department of Chemical and Biomolecular Engineering, College of Engineering, The Ohio State University, Columbus, OH 43210, USA; 4 Department of Biomedical Engineering, College of Engineering, The Ohio State University, Columbus, OH 43210, USA; 5 Department of Neuroscience, College of Arts and Sciences and College of Medicine, The Ohio State University, Columbus, OH 43210, USA; 6 School of Health and Rehabilitation Sciences, College of Medicine, The Ohio State University, Columbus, OH 43210, USA

**Keywords:** microfluidics, polymerase chain reaction (PCR), loop-mediated isothermal amplification (LAMP), raman spectroscopy, matrix-assisted laser deposition/ionization mass spectroscopy (MALDI-ToF MS), bacterial identification

## Abstract

Bacterial infections continue to pose serious public health challenges. Though anti-bacterial therapeutics are effective remedies for treating these infections, the emergence of antibiotic resistance has imposed new challenges to treatment. Often, there is a delay in prescribing antibiotics at initial symptom presentation as it can be challenging to clinically differentiate bacterial infections from other organisms (e.g., viruses) causing infection. Moreover, bacterial infections can arise from food, water, or other sources. These challenges have demonstrated the need for rapid identification of bacteria in liquids, food, clinical spaces, and other environments. Conventional methods of bacterial identification rely on culture-based approaches which require long processing times and higher pathogen concentration thresholds. In the past few years, microfluidic devices paired with various bacterial identification methods have garnered attention for addressing the limitations of conventional methods and demonstrating feasibility for rapid bacterial identification with lower biomass thresholds. However, such culture-free methods often require integration of multiple steps from sample preparation to measurement. Research interest in using microfluidic methods for bacterial identification is growing; therefore, this review article is a summary of current advancements in this field with a focus on comparing the efficacy of polymerase chain reaction (PCR), loop-mediated isothermal amplification (LAMP), and emerging spectroscopic methods.

## 1. Introduction

The Centers for Disease Control (CDC) estimates that in the Unites States 9.4 million people are infected with foodborne illnesses annually, with over 128,000 hospitalizations and 3000 deaths [1]. These numbers illustrate the risks pathogen infections pose to human health and therefore, constitute a serious public health concern [2,3]. Therapeutics such as antibiotics, antibody therapy, and vaccinations for treatment of pathogenic infections exist to alleviate many of these illnesses [4]; however, as microorganisms continue to evolve, the concern for resistance against these treatments continues to grow [5]. Early identification of disease-causing organisms becomes necessary to mitigate the spread of disease and initiate appropriate antimicrobial therapy [6]. Among the various pathogens such as viruses, bacteria, parasites, and fungi, methods for identifying bacteria to form a diagnosis of infection or knowing the organism causing contamination has been of significant focus due to the widespread prevalence [7] of bacteria across food, water, animal, or human interactions. Given the subsequent impact on animal and human health, the primary focus of much research has been on understanding the biology of the pathogens. However, engineered systems play a critical role in advancing biology and societal impact. In this review article, we emphasize the role of engineered systems driven by specific operating multi-physics that allow improved understanding of biology or provide translational advances in bacterial identification.

Among the engineered systems relevant to bacterial identification, the field of microfluidics finds a unique place. For example, microfluidic devices have been employed in clinical diagnostics, chemical and biological analyses, food and chemical processing and environmental uses, including mineral processing [8,9,10]. The use of laminar, low Reynolds number flows with small volumes permits many advantages that have been discussed in previous publications including many reviews [11,12,13,14,15].

The focus for this article is on the use of microfluidic devices coupled to additional techniques for the identification of bacteria in liquids. We note that detection of bacteria, which determines presence or absence of bacteria, is not a focus of this article as the field of biosensing is well reported upon [16,17,18]. Currently, the standard for bacterial identification relies on conventional methods such as semi-quantitative plate culturing combining Gram staining, culturing, and biochemical analysis [3], which require high concentrations (>10^3^ CFU/ mL) [19] of bacteria with 2–3 days to effectively process for identification [3,20]. Additionally, only 1% of known bacteria are considered culturable [21,22]. Difficulties differentiating bacteria have been previously observed among conventional approaches that utilize API (Analytical Profile Index) as this approach may lack specificity at the species and strain levels [20,23]. In the past two decades, overcoming the challenges to reduce detection time and biomass concentration, increase sensitivity and specificity, and eliminate false positives has been a focus of research. Notably, the objective to move away from culture-based methods to identify bacterial organisms and further analyze the specific strain has provided a challenge for the physical sciences community to develop new and emerging techniques, which are discussed in this article.

In microfluidics, lab-on-a-chip (LoC) devices present a potential solution to such challenges by enabling dramatically improved limits of detection with increased sensitivity, specificity, reduced sample volumes, and rapid detection times [24]. These devices can also enable point-of-care (PoC) diagnostics by integrating microfluidic channels with multiple aspects of biological and chemical processing into one device [24,25]. The reduced volume enabled by the use of microfluidic and nanofluidics-based LoC devices also provides an opportunity to develop culture-free bacterial identification methods.

**Figure 1:**
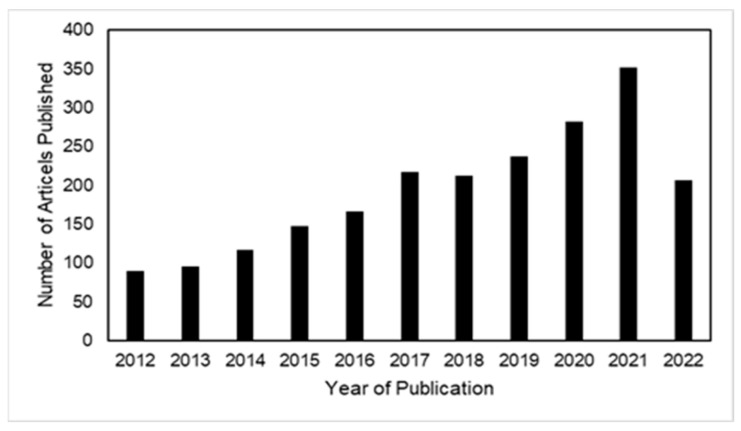
The figure indicates a growing interest in microfluidics-enabled devices for bacterial identification over the past decade by using the metric of number of research articles published over the last decade with keywords “microfluidics” and “bacterial identification”. Data obtained from Scopus using a keyword search.

We begin this mini review by demonstrating a growing interest in leveraging microfluidics for applications in bacterial identification. The growing interest is evidenced by the number of search results one encounters in Elsevier’s database Scopus using the search term “microfluidics” in conjunction with “bacterial identification”. The rising trend in the field over the last decade is depicted in Figure 1 with the last full year of 2021 showing nearly 350 published articles which represents nearly a 3-fold increase in published articles over the past decade.

Previous literature reviews have addressed the progress of microfluidics for bacteria identification via isolation and subsequent detection of nucleic acids, proteins and enzymes, and cells [26,27,28,29,30,31,32]. Nucleic acid-based devices conventionally utilize polymerase chain reaction (PCR) or microarray technologies to analyze DNA or RNA sequences with high sensitivity and specificity [33]. In contrast, protein or enzyme-based technologies rely upon binding affinities through which proteins interact and can be isolated via protein binding to the surface of the chip. These methods specifically target antigen–antibody binding, limiting use of such devices. Finally, cell identification has been another area of interest for bacterial identification without sensing sub-cellular components such as proteins.

Emerging methods that do not use culture-based techniques but rely on microfluidics combined with either amplification methods (e.g., PCR) or chemical spectroscopy (e.g., Raman spectroscopy) are summarized in Table 1. This article, in contrast to previous reviews, will focus on emerging methods and updates to existing methods. Therefore, the focus is on PCR (polymerase chain reaction), LAMP (loop-mediated isothermal amplification), MALDI-ToF MS (matrix-assisted laser deposition/ionization mass spectroscopy), and Raman spectroscopy to target bacteria, limits of detection, reaction times, and sample inputs and volumes, as summarized in Table 1. Most publications reference their limit of detection (LoD) as the minimum concentration at which the target specimen of the sample can be measured accurately. In the context of LoD, microfluidic devices provide an advantage due to the small volumes (generally, <1 nL) to achieve lower LoDs.

Therefore, the purpose of this review is to describe the physical principles that enable the use of microfluidics for bacterial identification using emerging techniques for a myriad of bacterial measurements and data analysis methods such as machine learning that likely enhance the utility of experimental observations.

## 2. Enabling Microfluidics

Microfluidics refers to the study of fluid dynamics within a fluidic conduit with critical dimensions of 100 µm or less, with common fluid volumes ranging from 10^−9^ to 10^−18^ L [48]. A wide range of materials have been reported for microfluidics-based devices, such as glass [49], silicon [50,51], quartz [52,53], paper [54,55], 3D printing material [56,57], epoxy resins, and polydimethylsiloxane (PDMS) [58,59,60]. PDMS is now one of the most used materials for LoC devices dedicated to pathogen identification because of their biocompatibility, ease of fabrication, low cost, high elasticity, and non-toxicity [31,61].

Bacterial identification using microfluidics often requires the ability to isolate or concentrate either the bacterial cells or another related analyte, presenting a unique identifier of the bacterial organism. Most often, the large surface area to volume ratios in microfluidic devices allow the surface chemistry and the flow dynamics to be leveraged to separate and isolate small particles prior to a bacterial measurement, creating an integrated, multi-physics approach. Therefore, we begin a technical discussion of exploiting flow physics that has found significant interest in the bacterial isolation and concentration domains.

Ease of use and operation has given significant prominence to the use of inertial flow to drive passive, size-based separation of particles in a heterogeneous solution. Moreover, the utilization of inertial microfluidics has grown in popularity due to its simple design and cost-efficient fabrication method [62]. Inertial-based systems have also garnered attention for the label-free isolation mechanisms and adaptability to varying reactor sizes and cell types.

Generally, these inertial microfluidic devices utilize either straight or curved channels to achieve desired separation of particles. In conventional microscale flows, a key parameter is the Reynolds number, Re, which compares the effect of inertial forces to viscous forces within a flow. Generally, in microfluidic and nanofluidic systems, laminar flow with Re < 100 is observed; however, depending on specific flow conditions, Re << 1 can occur, and these flows are referred to as creeping flows. In cases that these flows contain particles, conventional fluid mechanics dictates that cross-streamline migration of particles is prohibited as noted by the symmetry arguments arising from the viscous-dominated, linearized Navier-Stokes equations. However, it was discovered that for weakly inertial flows, i.e., Re~*O*(0.1), small, confined particles (a/H << 1; a being the particle diameter and H being the critical dimension for the microchannel) experience a lift force, permitting lateral particle migration. Initial descriptions of inertial lift forces date back to work by Segre and Silberg [63] and later by Bretherton [64]. With progress in microfluidics, the wall-induced lift force may be used to initiate particle-size-based migration and focusing to achieve particle isolation [65].

**Figure 2:**
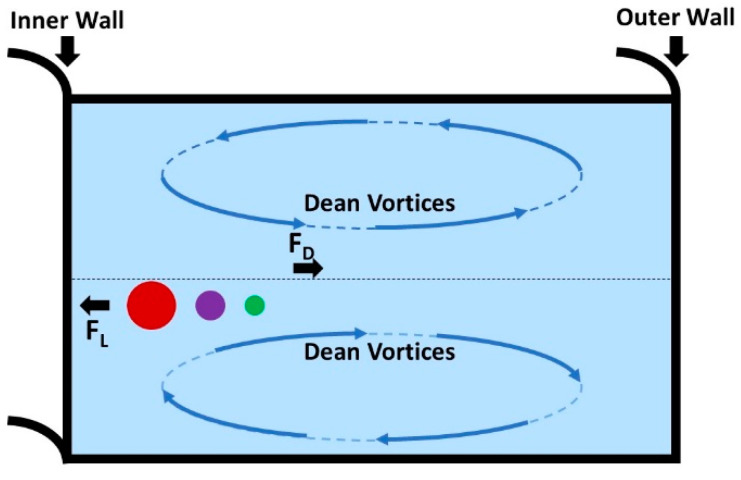
Cross-sectional schematic of a curvilinear microchannel showing the generation of Dean vortices and the forces that act on particles to cause lateral migration in low Re flows, along with inertial lift force and drag forces which affect positional equilibrium of particles within the microchannel. Adapted from M.-L. Lee and D.-J. Yao, *Inventions* 2018, 3, 40; licensed under a Creative Commons Attribution (CC BY) license.

Continued progress in microfabrication allowed the use of curved microchannels, where in addition to the inertial lift force, the curvature of the microchannel could also be exploited. The curvilinear geometry of the channels subjects the fluid to a radially outward-directed centrifugal acceleration. This effect enables a secondary flow, known as Dean flow, that results in two counter-rotating Dean vortices that are positioned symmetrically about the channel centerline as depicted schematically in Figure 2. The curvature of the channel and these induced vortices are often quantified by the Dean number (De) shown in Equation (1), where  Re is the Reynolds number, $D_{h}$ is the hydraulic diameter, and r is the radius of the channel curvature [66,67]. The Dean number presents a ratio not only of the inertial to viscous forces in the flow, but the curvature ratio accounts for the centripetal effects too.


(1) $$De=Re\sqrt{\frac{D_{h}}{2r}}$$

The Reynolds number is defined by Equation (2):

(2) $$Re=\frac{\rho U_{m}D_{h}}{\mu }$$

where ρ is the fluid density, $U_{m}$ is the mean fluid velocity, and μ is the fluid viscosity [66]. Dean vortices influence where particles are positioned within the channels, helping establish a positional equilibrium. Additionally, the location of particles within a microchannel is also affected by the interplay between opposing inertial lift forces, $F_{L}$ (shown in Equation (3)), and drag forces, $F_{D}$ (shown in Equation (4)).

(3) $$F_{L}=\rho {\left(\frac{U_{m}}{D_{h}}\right)}^{2}C_{L}a_{p}^{4}$$

(4) $$F_{D}=5.4\times 10^{-4}\pi \mu De^{1.63}a_{p}$$

$C_{L}$ is the lift force coefficient and $a_{p}$ is the size of the particle. It can be noted from Equations (3) and (4) that when the size of the particle increases, the inertial lift forces start to dominate, resulting in particles migrating and equilibrating away from the outer wall (following the direction of the Dean vortices) and towards the inner wall of the microchannel [66,68]. These operating physical principles were utilized in the separation of larger *Saccharomyces pastorianus* from smaller *Lactobacillus brevis* within the microfluidic channel design from Condina et al. [68]. Post-separation, the work by Condina et al. deployed matrix-assisted laser desorption/ionization time-of-flight mass spectrometry (MALDI-ToF MS) to achieve an accurate identification of bacteria. Therefore, microfluidics enabled use of a combinatorial technique for bacterial identification without use of culture-based methods.

### 2.1. Microfluidics with PCR

As described in the previous section, flow physics can be exploited to generate viable particle isolation to achieve bacterial identification in samples with mixed populations. A common method used for profiling and identifying bacteria relies on polymerase chain reaction (PCR). PCR is an amplification method that can be used in bacterial identification. Several million copies of the DNA can be produced from a single molecule of the sample by several cycles of the PCR. A PCR cycle typically consists of three temperature-dependent steps, namely, denaturing, annealing, and extension [69,70,71,72]. During the denaturation phase, the genetic material containing sample is heated above its respective boiling point (95 °C) to ensure uncoiling and separation of DNA strands [71,72]. Subsequently, during the annealing phase, the temperature is lowered (55 °C) to enable attachment of specific primers to target DNA segments [72]. Finally, the targeted and primed segments are extended during the extension phase where the temperature is raised again (72 °C) [72]. Traditional PCR systems require 20–30 cycles to amplify DNA. To facilitate faster PCR, continuous flow PCR (CF-PCR) has been reported. The miniaturization of the PCR process via microfluidics addresses shortcomings such as bulky instrumentation and large reaction volumes experienced by conventional PCR methods [73]. The fabrication of a portable CF-PCR system makes it possible to conduct testing in locations where rapid identification is necessary with minimal equipment storage [74,75]. The importance of temperature and process control and virtues of PCR technologies have been extolled in many previous reviews [76,77], and the reader is referred to those articles for further information on this technique.

The emerging use of microfluidics to enable new discoveries by utilizing either on-chip PCR or microfluidics to concentrate and extract high quality nucleic acid for use in standard PCR has provided significant advances. For example, in the recent work by Hoamann et al. [78], a microfluidic cartridge provided an integrated on-chip platform for reagents and automated the collection of bacteria, bacterial lysis, and subsequent extraction of nucleic acids. They demonstrated the potential of their system for diagnosis of *Mycobacterium tuberculosis*. This work by Hoamann et al. exploited the microfluidic channel geometry laid out in a radial pattern such that the sample (sputum) collection chamber with distinct chambers for reagents and bacterial filters can be placed in a commercial centrifuge. The bacterial separation exploits the flow-physics where the centrifugal pressure of the liquid within the device acts on an outer seal to allow transport of liquids and mixing of reagents. One advantage of exploiting microscale fluid physics is the ability to develop workflows that allow integration of sample preparation systems with existing equipment and enable rapid identification of bacteria relevant to critical human diseases. Notably, these systems can also be used for environmental monitoring but have found limited use in such applications.

Similarly, CF-PCR systems have proven useful for various pathogenic bacteria [79,80,81]. Huang et al. [79] discussed the results of a microfluidic chip-based PCR-array system, Onestart, in which the reagents required for PCR amplification were included on the chip to create a ready-to-use device that can complete detection of 21 respiratory tract bacterial pathogens. Experimentation discovered the entire process can be completed within 1.5 h with a limit of detection of 1.0 × 10^3^ genetic copies/mL at 100% specificity [79].

In another demonstration, Bae et al. [80] fabricated a disposable film-based PCR chip containing a multiplexed gene chamber from polyethylene terephthalate (PET) and polyvinyl chloride (PVC) adhesive film that enabled simultaneous gene amplification. The multi-layer structure comprising distinct polymer layers necessitated the characterization of the temperature profiles to ensure that the PCR-process was not impacted by the microfluidic chip design. Such characterization shows that for these microfluidic devices, considerations for design need to pay attention to not only material and process chemistry but also operating physical principles that govern PCR. Three different chips with critical dimensions (230 µm, 460 µm, and 920 µm respectively) were used for bacterial detection in samples of milk artificially infected with various concentrations of *Bacillus cereus*. The 230 µm chip demonstrated the best PCR performance with a 1.0 × 10^1^ CFU/mL limit of detection and exhibited feasibility for point-of-use testing [80].

Microfluidics, as noted previously, permits use of multiple physical phenomena for flow manipulations. Therefore, recognizing that most biological specimens, including bacteria, possess a finite electrical charge has promoted the use of electrokinetic phenomena which has found use along with standard flow-physics for microfluidic advancements. Among the various electrokinetic phenomena, the approach of using dielectrophoresis has been demonstrated for bacterial separations to enable downstream identification in microfluidics. Dielectrophoresis is an electrokinetic phenomenon in which dielectric particles (e.g., bacterial cells) can experience a force in the presence of a non-uniform electric field. Inherent to dielectrophoretics is also the idea that for dielectric materials, the particle itself may not be charged. The difference in the dielectric properties of the particle with respect to the surrounding fluid is critical to the implementation of dielectrophoresis [82].

Use of dielectrophoresis for separation of bacteria at a strain level has been shown to minimize auxiliary equipment and therefore simplify the operating workflow [83]. Additionally, other electrokinetic phenomena that exploit use of charge-to-mass ratio for separations have also been used. For example, Li et al. [84] fabricated a portable all-in-one microfluidic device that integrates CF-PCR with an electrophoresis in a serpentine microchannel. This serpentine geometry increases the total length of the channel and allows for sufficient dwell times in the channel for the three phases of PCR [84]. Designed for rapid detection of pathogens, the device was assessed on the detection of three periodontal pathogens (*Porphyromonas gingivalis*, *Treponema denticola* and *Tannerela forsythia*) where amplification occurred in under 3 min and detection occurred in under 4 min with a minimum amplification of 125 CFU/mL [84]. Though the device offered a mechanism to successfully amplify more than one target gene on the chip, it was suggested by the authors that cross-reactions may have resulted in false positives.

**Figure 3:**
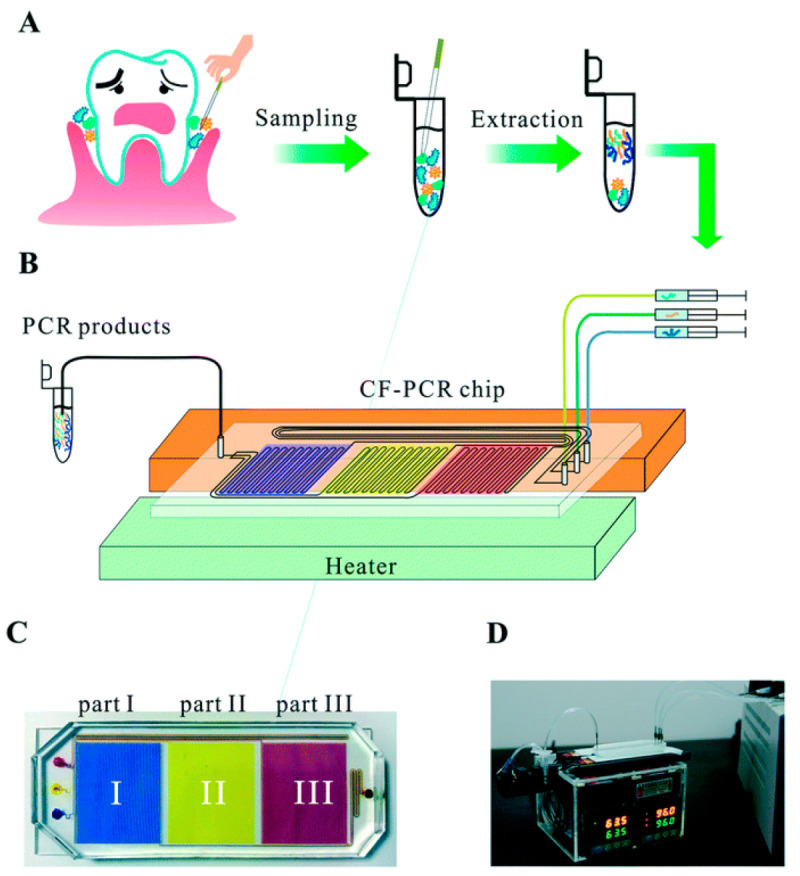
Example depiction of CF-PCR-based chip. (**A**) General sampling and extraction methods used for obtaining experimental periodontal pathogens tested for identification using the device. (**B**) Diagram of the microfluidic chip containing serpentine channels mixing the PCR reagents with the extracted sample above a thermocycler heating system. (**C**) Explicit definition of the three divisions and stages of the device. (**D**) Overall image of the device. Reproduced with permission *Lab on a Chip*, 2022, 22, 733–737. Copyright 2022 Royal Society of Chemistry.

The possibility of false positives was alleviated by Yang et al. [85] (Figure 3) by dividing the device to three parts to amplify target genes of periodontal pathogens *Porphyromonas gingivalis*, *Tannerella forsythia*, and *Treponema denticola* separately but still on the same chip. Each targeted gene had individual amplification times of ~2 min, ~3 min, and ~5.5 min, respectively, with a simultaneous time ~8 min [85]. The use of the three zones showed the ability to minimize cross-reactions and reduce false positives; however, the fundamental question of why such an effect was observed remained unanswered. One of the issues that remains unresolved for CF-PCR, despite the many advances, is the appropriate thermal isolation needed for each step, especially when isolating and amplifying from a sample containing a mixture of bacteria. Therefore, in the domain of multi-physics microfluidics, advances remain to be achieved in device design and implementation. Nevertheless, recent advancements, including many translational achievements, suggest feasibility for multi-physics-enabled microfluidic devices.

Traditional PCR is unable to differentiate between live and dead cells, i.e., the viability of bacteria, as PCR offers broad specificity and the amplification of DNA sequences originating from both live and dead cells, leading to the possibility of false positive detection and unrepresentative results. To address this concern, Zhu et al. [86] fabricated a serpentine microfluidic channel that incorporates an on-chip propidium monoazide (PMA) pre-treatment to tag viable bacteria during the annealing phase of PCR cycle [86]. In this work by Zhu et al., the use of biochemistry was needed as a pre-treatment to enable advances in use of PCR. Notably, for microfluidic devices, often a modular design is used that allows a staged series of operations with multiple reagents, reactions, and distinct unit operations to be performed.

In a different use of this technology, environmental samples were targeted at a flow rate of 35 mL/min. Pond water was used to test the efficacy of the design and demonstrated considerable success when differentiating between live and dead cells with potential to extend this method for bacterial identification. Additionally, Madadelahi et al. [87] contributed an advancement to PCR amplification with their design of a microfluidic chip that combined a serpentine channel, which served the function of droplet generation and reagent mixing via transient secondary flows, with a 27-cycle spiral channel that allowed for sufficient thermal cycling to reduce PCR time by ~40% compared to previous methods. The authors incorporated diamond nanoparticles (diamondNP) into the PCR solution to enhance PCR performance more than five times depending on diamondNP concentration [87]. As shown throughout this section, multi-physics-based devices that combine design features, thermal cycling, flow physics, and electrokinetic phenomena continue to show advances in identification of bacteria.

### 2.2. Microfluidics with LAMP

Like PCR, loop-mediated isothermal amplification (LAMP) requires extraction of nucleic acids for amplification. LAMP technology is a recent advancement in the field of rapid pathogen detection in which a method of auto-cycling strand displacement DNA synthesis is utilized in the presence of Bst DNA polymerase under isothermal conditions between 60 and 65 °C [88,89]. Within 30–60 min, this method has the ability to produce 10^6^–10^9^ copies of targeted DNA strands. Even though, like PCR, LAMP is also a thermally mediated amplification process, it occurs isothermally at a single temperature and does not require multiple temperature stages like PCR. As a newer technology, LAMP offers the advantage of lower cost and higher operating speed than PCR. Moreover, unlike PCR amplification methods, LAMP technologies offer advantages for point-of-care testing in that they do not require a complex thermocycler to function [90,91].

The LAMP process consists of two distinct phases—the initial phase and cyclic amplification where in total, four primers (two inner and two outer) are used to enhance strand displacement activity [88,92]. During the initial phase, all four of the primers are used, while only the two inner primers are used during the latter [92]. Overall, the reaction results in the DNA being coiled into a stem-loop with numerous inverted target repeats that can be subsequently identified using fluorescence spectroscopy [92] or a digital reader [93]. The advance in biophysics due to LAMP methods can now be coupled to microfluidics for further advancements. Recently, LAMP technologies have proven useful in microfluidic platforms for rapid pathogen detection [94] with some examples comparing LAMP and PCR methods in terms of copies produced, time of detection, and sample volume summarized in Table 1.

**Figure 4:**
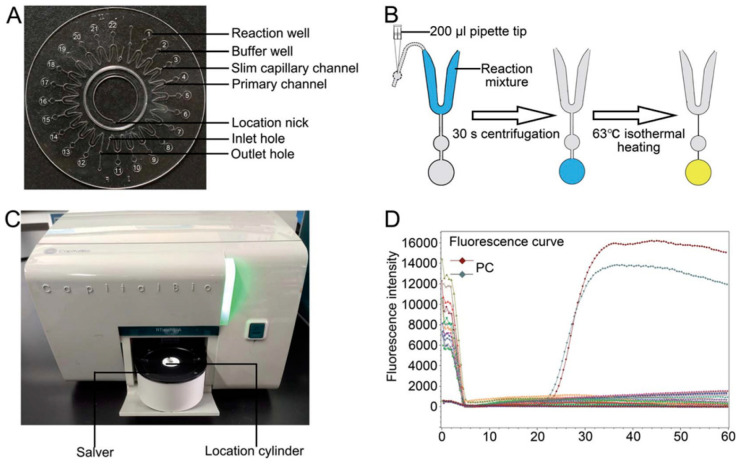
LAMP-based dual-sample microfluidic chip that allow for simultaneous amplification and real-time detection of genetic material from 10 waterborne pathogens. (**A**) Image of the dual-sample microfluidic chip that consist of two identical half units containing 11 reaction wells each. (**B**) Methodology of sample amplification and path of reaction mixture flow through channel. At 63 °C, their slim capillary channels narrow and fuse together, preventing contamination of reaction mixture. (**C**) CapitalBio RTisochip-A apparatus used for isothermal heating fluorescent acquisition. (**D**) Fluorescent plot with time to positive amplification on the x-axis and intensity of fluorescence on the y-axis. Reproduced with permission from *Analytical Methods* 2021, 13, 2710–2721. Copyright 2021 Royal Society of Chemistry.

Due to the isothermal operation of LAMP and reduced need for auxiliary equipment, LAMP techniques have found applications beyond human or animal health-related clinical applications. Notably, as summarized in Table 1, bacteria such as *Escherichia coli* and *Salmonella enterica* are also identified as waterborne pathogens making them capable of contaminating food easier than other forms of bacteria. Biosensors for food or waterborne bacteria largely focus on tracking the concentration and activity of these analytes [95]. Use of real-time LAMP to identify bacterial species remains an open question. Nevertheless, LAMP in conjunction with microfluidics has proven useful in detection of waterborne pathogens. Jin et al. [96] demonstrated rapid detection of waterborne bacteria using their LAMP-based dual-sample microfluidic chip (Figure 4) in which the genetic material of 10 pathogens (*Campylobacter jejuni*, *Listeria monocytogenes*, *Salmonella enterica*, *Shigella flexneri*, *Staphylococcus aureus*, *Vibrio alginolyticus*, *Vibrio cholerae*, *Vibrio parahemolyticus*, *Vibrio vulnificus*, *and Yersinia enterocolitica*) were simultaneously amplified and analyzed for detection from two specimens. At a run-time of 35 min, the device achieved limits of detection ranging from 7.92 × 10^−3^ to 9.54 × 10^−1^ pg of DNA/ reaction with 93.1% sensitivity and 98.0% specificity [96]. The advancement was enabled using a disk-like microfluidic device, once again utilizing centripetal forces for manipulating the bacteria-flow interactions. Moreover, the use of microfluidics through multiple chambers allowed automation in reagent interactions, permitting use of techniques like LAMP and an overall reduction in operation time. Therefore, combining engineering principles with flow physics provided an avenue for translational advance in identification of waterborne bacteria.

In another report, Jiang et al. [97] identified airborne bacterial strains through the use of a high-throughput microfluidic device to analyze the presence of five types of bacteria (*Staphylococcus aureus*, *Escherichia coli*, *Pseudomonas aeruginosa*, *Citrobacter koseri*, and *Klebsiella pneumonia*) in bioaerosols. The microfluidic device consisted of five chambers and channels pre-coated with LAMP primers specific to each type of bacteria in which the sample flowed into the chambers and the DNA specific to that primer was amplified to determine the bacterium’s presence. Each chamber demonstrated specificity to its corresponding bacterium, and *Staphylococcus aureus* was used to determine a detection limit of 24 cells per reaction so that results could be observed by the naked eye [97].

Another example of the utility of LAMP with microfluidics was demonstrated by Zhou et al. [94] in which a reverse transcription LAMP (RT-LAMP) with four independent units that each consist of eight reaction wells was used for simultaneous amplification and detection of three porcine enteric coronaviruses: porcine epidemic diarrhea virus (PEDV), porcine deltacoronavirus (PDCoV), and swine acute diarrhea syndrome-coronavirus (SADS-CoV). The diagnostic chip was able to provide sensitivities of 92.24%, 92.19%, and 91.23% for PEDV, PDCoV, and SADS-CoV, respectively, at 100% specificity with a 40 min run-time and limits of detection of 10^1^ copies/µL, 10^2^ copies/µL, and 10^2^ copies/µL for PEDV, PDCoV, and SADS-CoV, respectively [94].

Notably, the advances described in this section were enabled by use of disk-like microfluidic devices that, as described previously, use centripetal forces for manipulating the bacteria-flow interactions. Moreover, the use of microfluidics through multiple chambers allowed automation in reagent interactions permitting use of techniques such as LAMP and an overall reduction in operation time.

### 2.3. Microfluidics with Mass Spectrometry

Both PCR and LAMP methods have provided major advances in bacterial identification, yet both techniques require nucleic acid extraction which can introduce errors. Therefore, erroneous outcomes due to sample handling and processing are possible in either method. On the other hand, analytical chemistry has provided tools to avoid such errors. One of such techniques is matrix-assisted laser desorption/ionization-time-of-flight mass spectrometry (MALDI-ToF MS). Mass spectrometry is an analytical technique that quantifies the mass-to-charge ratio of the molecules present within a sample. As noted in a previous review on MALDI-ToF MS, the interest in the microbial community to use this technique for bacterial identification started gaining interest in the 1980s due to advancements in electron spray ionization and matrix-assisted laser desorption ionization (MALDI).

Now, MALDI-ToF MS is a technology used in laboratory settings for pathogen and cell identification [98,99,100,101,102,103]. The MALDI-ToF MS spectrum from a microbial sample provides a unique signature for specific identification at the species level [98]. The sample must first be coated with a matrix containing an energy-absorbent organic compound whereupon drying, both the sample and matrix will crystallize [6,100]. Upon desorption and ionization with a laser beam, singly protonated ions are formed from the sample and can be accelerated to determine their mass-to-charge ratio (*m*/*q*) [6]. Subsequently, in time-of-flight (TOF) technologies the *m*/*q* is measured based on the amount of time required for the ion to travel the length of the flight tube [6,100]. Based on this information, a peptide mass fingerprint (PMF) can be formulated and used to specify the microorganism within the sample [6].

**Figure 5:**
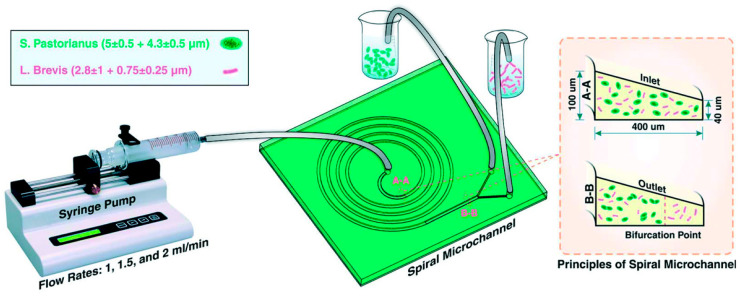
Graphic representation of the experimental workflow of the inertial separation spiral microfluidic device created by Condina et al., where it can be seen that larger *Saccharomyces pastorianus* position near the inner channel wall and smaller *Lactobacillus brevis* concentrate at the outer wall. Reproduced with permission from *Lab on a Chip*. 2019, 19, 1961–1970. Copyright 2019 Royal Society of Chemistry.

As discussed briefly in the section on enabling microfluidics, Condina et al. [68] addressed brewery contamination concerns with a device that includes spiral microchannels combined with MALDI-ToF MS for inertial separation and identification of two types of beer spoilage microorganisms (*Lactobacillus brevis* and *Pediococcus damnosus*) from yeasts (*Saccharomyces pastorianus* and *Saccharomyces cerevisiae*). In their work, they first used inertial separation to collect bacteria before the MALDI-ToF MS analysis to increase the sensitivity of their measurements. At an optimized flow rate of 1.5 mL/min, the spiral microfluidic channel achieved 90% efficiency of *Lactobacillus brevis* separation from *Saccharomyces pastorianus*. Subsequently, the isolated microorganism mixture was processed through a traditional MALDI-ToF MS system off-chip where it detected the pathogen with high accuracy [68]. Using the inertial microfluidic separation, it addressed the issue of confounding MALDI-ToF MS spectra overlap caused by the presence of multiple bacteria in a sample, thereby improving the limit of detection with the MALDI Biotyper without requiring bacterial cultivation [68]. Therefore, the use of microchannel design to manipulate flow physics and combining with an emerging mass spectrometry technique provides another example of using multi-physics phenomena for bacterial identification. The limit of detection after inertial separation improved from 1 colony/2 mL to 1 colony/4 mL for *Lactobacillus brevis* and from 3 colonies/mL to 1 colony/mL for *Pediococcus damnosus* [68]. The device used in this work is shown schematically in Figure 5 wherein the right panel depicts the use of flow physics to improve separations and provide higher efficacy for the MALDI-ToF MS technique.

**Figure 6:**
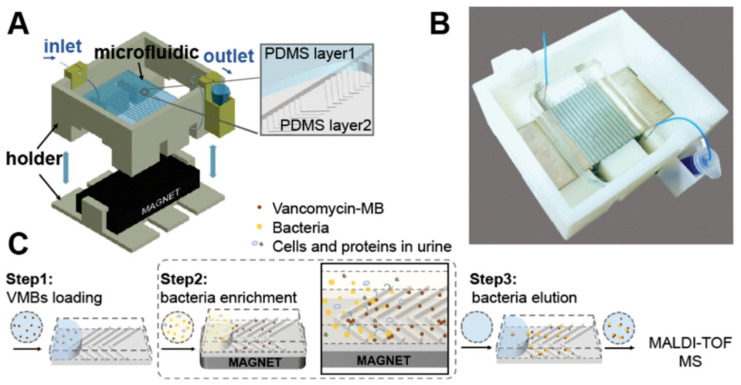
Microfluidic chip utilizing a herringbone design and vancomycin modified magnetic beads (VMB) to enrich bacteria for MALDI-ToF identification. The herringbone design aids in generating chaotic flow for more efficient mixing and interaction of VMBs and bacteria. (**A**) Microfluidic model assembly displaying the staggered herringbone design. (**B**) Image of assembled microfluidic setup and its serpentine channels. (**C**) Work process for pathogen identification. Reproduced with permission from *Analyst*. 2021, 146, 4146–4153. Copyright 2021 Royal Society of Chemistry.

The reduced volumes that have provided key advantages to microfluidic technologies also pose a challenge. For mass spectrometry, adequate biomass is needed to ionize the sample for a viable measurement. As sample volumes decrease, so does the available biomass. To tackle the limitations of biomass requirements for accurate identification, Shen et al. [43] utilize a herringbone microfluidic chip (Figure 6) incorporating vancomycin modified magnetic beads to achieve more efficient enrichment of four types of bacteria (*Staphylococcus aureus*, *Staphylococcus hominis*, *Staphylococcus epidermidis*, and *Enterococcus gallinarum*) from urine samples. The herringbone design allows the mixing of contents within the microfluidic channel, which would not occur in straight channels as the low Re flows are laminar. The herringbone design has been a common tool for microfluidics researchers to improve mixing and significant past reporting has occurred on this topic, and therefore the specifics of the design are not discussed here. However, the reader is referred to the past literature for further reading [104,105].

With improved mixing, interactions between pathogens and vancomycin modified magnetic beads were enhanced [43]. The device achieved an enhanced 90% capture efficiency of bacteria in which the resulting sample underwent MALDI-ToF MS off-chip to identify the bacteria with a limit of detection of 10^4^ CFU/mL for *Staphylococcus aureus*, *Staphylococcus hominis*, and *Staphylococcus epidermidis* and 10^5^ CFU/mL for *Enterococcus gallinarum* [43]. As in these two papers, most microfluidic systems require manual sample transfer to a MALDI plate in order to perform MALDI-ToF MS, which creates difficulties when attempting to create an all-in-one separation and identification device [101].

Many previous reports have used the strategy of integrating multiple chambers or stages in the microfluidic devices. Similarly, Li et al. [44] attempted to avoid sample transfer by the use of a silicon nanowire for capturing *Escherichia* *coli* from urine samples and directly administering MALDI-ToF MS to the nanowire. In such an approach, another emerging advance beyond the scope of this review article is observed. The integration of distinct materials and fabrication technologies on a single device provides another avenue for engineering advances. The device demonstrated accuracy in enriching and identifying the bacteria in under an hour at a concentration of 10^6^ CFU/mL for uncultured samples and at a concentration of 10^3^ CFU/mL for samples that were cultured for 4–6 h [44]. Though the device was able to achieve pathogen identification without manually transferring the sample, limitations regarding the efficiency of MALDI-ToF MS in identifying microorganisms at low concentrations and rapid times remained for scenarios where the sample quantity may be limited.

## 3. Raman Spectroscopy Based Methods

Previous methods of PCR, LAMP, or MALDI-ToF MS require extensive sample preparation to achieve adequate purity for sample manipulation. On the other hand, chemical spectroscopic techniques have been used for many decades to identify signature molecules in the chemical and physical sciences. One such method is Raman spectroscopy. In this method, light is used to analyze samples through the inelastic scattering of photons resulting from light-sample interactions. Most of the scattered photons will have the frequency of the incident radiation and can be categorized as Rayleigh scattering. The smaller division of scattered photons differs in frequency and is known as Raman scattering [106]. The difference in frequency allows the measurement of energy between the incident and inelastic photons, providing a unique molecular vibration fingerprint [107]. When Raman scattering occurs at a lower frequency than Rayleigh scattering, Stokes lines are present on the Raman spectrum. When the opposite is true, anti-Stokes lines are present [106]. The Raman spectra can be used to qualitatively and quantitively classify samples. Additionally, Raman spectroscopy is non-destructive to samples during the spectra retrieval, making it especially useful for the investigation of biological samples.

Raman spectra of living cells can be acquired in real-time without disturbing them in addition to acquisition in confined spaces. Unlike in infrared spectroscopic analyses, water does not significantly affect the quality of signal, making the analysis of bio-fluids possible [108]. However, Raman spectroscopy brings its own challenges, specifically, low efficiency of Raman scattering due to the scattering cross-sections ranging from 10^−29^ cm^2^ to 10^−21^ cm^2^ per molecule [109]. These scattering cross-sections are much smaller than those for typical infrared spectroscopy, making the Raman measurements harder. Subsequently, different techniques have been developed to enhance the Raman scattering effect. One such technique is surface-enhanced Raman spectroscopy (SERS) where plasmonic interactions with metallic substrates enhance Raman signal intensity up to 14 orders of magnitude higher than conventional Raman. Common substrates include immobilized metal nanoparticles (NPs) and metallic NP colloids. This method of Raman decreases analysis time, increases sensitivity, and increases identification resolution [110]. There are some difficulties when it comes to using SERS such as matrix interference, but this limitation can be remedied through the use of microfluidics [111].

For example, Li et al. used SERS to identify and analyze drug sensitivity of blood pathogens, with the goal of accelerated blood infection diagnosis and knowledge of bacterial susceptibility. Bacteria acquired from blood samples (concentration of 10 CFU/mL to 10^3^ CFU/mL) were enriched by Fe_3_O_4_-PEI [112]. Next, the Fe_3_O_4_-PEI-bacteria complex was grown on both routine agar plates and drug sensitive plates. The difference in SERS signal was compared between single colonies’ SERS spectra and standardized bacterial spectra. Using OPLS-DA for discriminant analysis, it was determined that this method was successful in detecting and identifying all drug-sensitive and drug-resistant strains of bacteria in the 77 clinical blood infection samples in addition to other bacterial strains used to verify efficacy.

In another study, two SERS-based chips were designed using different materials—Al foil and A4 paper. To have both chips be SERS active, colloidal silver nanoparticles (AgNPs) were added to the detection area of the devices. After testing both devices using the Rhodamine 6G (R6G) molecule, it was determined that the Al foil-based chip allowed for better SERS performance. With that, bacterial identification using *Escherichia coli* and *Staphylococcus aureus* was tested in the device concluding a standard deviation of 12.5% to 11.7% between any 36 random detection points. Using the chip allowed for a short detection time and a high throughput [113].

In another LoC device, Hou et al. developed a technique to overcome the limitation of low bacterial concentrations in a sample. The device coupled a pre-concentration method, consisting of a discharge driven vortex that converges flow to a stagnation point, with on-chip detection using SERS. Once again, it is worth noting that the microfluidic device enabled the use of manipulation of flow physics to condition the sample appropriately prior to spectroscopic probing. Using dilute bioparticle samples as low as 10^4^ CFU/mL (in a standard 150 µL batch volume), the concentration vortex yielded a packed mound of bacteria within 15 min (width ~200 µm). These mounds, which consisted of either *Saccharomyces cerevisiae*, *Escherichia coli*, and *Bacillus subtilis*, had spectra taken before and after combination with silver nanoparticles. By producing unique signals among the bacteria and by illustrating distinct enhancement, SERS revealed that this technique was successful in producing characteristic spectra of low concentration bioparticle samples within 15 min [114].

Rodríguez-Lorenzo et al. [47] demonstrated real-time discrimination between the bacteria *Listeria monocytogenes* and *Listeria innocua* while undergoing continuous flow in under 2 min [47]. In another study, Dina et al. [115] used a microfluidic device integrating continuous flow and a silver-spot technique using SERS demonstrated rapid identification of four distinct bacterial species consisting of both Gram-positive and Gram-negative specimens in under 15 min [115].

While Raman spectroscopy is promising for bacterial identification, its application is hindered by the variable nature of bacterial growth due to different preparation conditions such as culturing temperature, growth media composition, and cultivation length [116]. These condition changes can produce metabolomic changes in bacteria that can modulate the spectral reading, thereby generating deviations from a consensus spectrum. Several studies that explored Raman-based identification of bacteria have consistently noted high intra-species variability in collected spectra, suggesting that slight variations in preparation conditions can interfere with accurate identification [117,118,119]. In addition, issues inherent to Raman spectroscopy such as weak signal, susceptibility to noise, and self-fluorescence can interfere with a coherent spectrum, despite the use of microfluidics to concentrate samples for maximum yields. However, while SERS allows for signal enhancement through the plasmonic interaction, inconsistent substrate nanostructures along with high background fluorescence reduce the signal-to-noise ratio, further impeding reproducibility of bacterial spectra [120].

To address these gaps in use of Raman spectroscopy, artificial intelligence (AI) has been used to develop machine learning models that can identify bacteria based on recorded spectra [121,122,123,124]. AI algorithms define patterns in data that are otherwise invisible to the human eye or lower-level statistical methods. Specifically, a subset of AI methods called supervised classifiers allow researchers to classify data into known classes. In the case of bacterial identification, supervised classifiers would read a spectral reading blinded to the bacterial species and would be able to identify that reading as originating from the known species. A supervised classifier would be able to do this through a pre-emptive “training phase”, where the naive classifier is given Raman spectra with the corresponding bacterial species and would learn from the underlying patterns, which spectral wavenumbers allow for the best differentiation between bacterial species. Next, we present a brief overview of these emerging AI methods for Raman spectroscopy. We note that these approaches have not yet been fully implemented with microfluidics. Yet, these methods have been shown to hold promise with limited implementation and therefore, merit a discussion in this article, whose main purpose is to highlight the coupling of microfluidics across domains to assist in identification of bacteria with non-culture-based techniques.

Numerous algorithms have been developed to classify data in a supervised manner such as random forests (RFs), support vector machines (SVMs), gradient boosted machines (GBMs), and k-nearest neighbors (KNN) classifiers [124]. In the context of bacterial spectral analysis, these algorithms show high accuracy across various tasks such as species vs. species or strain vs. strain classification. Unsupervised methods have been applied to differentiating between strain and pathogenicity but have underperformed in this regard [125]. An example of a supervised approach can be seen in Lorenz et al., who modeled an SVM that was able to differentiate pathogenic strains of *Escherichia coli* from non-pathogenic strains with 81.1% accuracy [126]. SVMs are exceptional classifiers that utilize hyperplane optimization to demarcate data from different groups [127]. With high-dimensional datasets, SVMs utilize a kernel function to map data into a higher dimensional feature space where data can be separated through linear means. The main drawback of SVM approaches is that they are designed for binary classification problems (A vs. B) as opposed to multi-class problems (A vs. B vs. C). Multi-class classification is a key goal in bacterial identification given the number of known species and strains, which makes an SVM approach to bacterial classification more difficult. SVMs can be adjusted to address multiclass problems using a one vs. all or one vs. one approach [128]. The one vs. all approach would require multiple SVM hyperplanes that ask whether a data point belongs to one class or not (A vs. all, B vs. all, C vs. all). On the other hand, the one vs. one approach creates classifiers between all possible binary combinations, (A vs. B, B vs. C, C vs. A), with the inferred class based on majority decision [129]. Several applications of multiclass SVM approaches to bacterial classification have shown success. One such application, done by Rahman et al., utilized the one vs. one strategy to classify spectra between 19 different bacterial strains, resulting in 87.9% accuracy [130]. As such, SVM algorithms have shown great potential to classify bacteria between binary and multiclass problems. A potential drawback of SVM algorithms as opposed to the other listed algorithms is there is an upper limit on the amount of data used in the training phase, as the training time scales steeply with the amount of data, specifically with a complexity of O(n^3^) [131]. In addition, the hyperparameters of the SVM algorithm (cost, kernel type, and gamma) have many possible combinations, which could possibly require extensive tuning to find the most optimal settings.

Along with SVMs, random forests (RFs) have been repeatedly applied to bacterial classification problems. RFs utilize multiple decision trees trained on random subsets of the overall dataset to determine the classification of data based on the average output of the decision trees [132]. The building blocks of RFs are decision trees, which classify data using specific thresholds based on features which best differentiate the data. Individual decision trees have a tendency of overfitting during the training phase, which can result in high training accuracy but low accuracy when “testing” the trained classifier on data not in the training set [126]. The ensemble approach of RFs, utilizing decision trees which have only seen a subset of the data, prevented overfitting and also allowed for robustness against noise. Kanno et al. opted to use RFs to classify spectra within a set of bacterial and archaeal strains achieving an accuracy of 98.9% [133]. Another application of RFs to bacterial Raman spectra was done by Zhang et al., who were not only able to classify cell type with high accuracy (91.6%), but were also able to identify the growth phase of bacterial (lag phase, log phase, stationary phase) with a sensitivity of 90.7% and a specificity of 90.8% [134]. One drawback of RFs is the loss of interpretability compared to decision trees. A single decision tree provides a consensus flowchart from which one can infer important features which allow for differentiation in a dataset. Inferring important features from multiple decision trees, numbering in the thousands, is much more difficult [134]. In addition, the number of trees one selects for their RF algorithm should be carefully considered as too many trees can making the training process tedious while too little trees can result in issues such as overfitting or noise susceptibility.

Another AI algorithm used in classification problems is gradient-boosted machines (GBMs). Given a base weak learner, (i.e., a decision tree, linear regression, splines, etc.), a gradient boosting approach would train the model on the overall dataset multiple times [135]. During each training phase, a loss function would be applied to the model, measuring the error or the cost associated with the model. Subsequently, the model is adjusted so that the gradient of the loss function reaches a minimum. This process of training, applying the loss function, and adjusting the model is repeated on the dataset to produce a refined model that surpasses the limitations inherent to the base weak learner. A variant of gradient-boosting, named stochastic gradient-boosting, utilizes the sampling approach of RFs such that each training phase uses a random subset of the overall data [136].

For classification of bacteria via Raman spectra, GBMs have shown promise. For example, Liu et al. applied a GBM, along with other AI algorithms, to classify between 15 strains of *Klebsiella pneumoniae* that were either resistant or susceptible to the drug Carbapenem [137]. The GBM, in this context, showed an accuracy of 99.40% when classifying data between resistant and susceptible type strains. Tang et al. also applied a GBM to classify different clinical species of *Staphylococcus*, with the GBM having an accuracy of 94.55% [138]. While GBMs have a strong level of performance in the discussed context, there are some disadvantages that should be addressed when using this method. The main consideration appears in training time as iterations to reduce the loss function in addition to the number base learners can take up large portions of RAM.

Lastly, k-nearest-neighbors (KNN) classifiers classify data based upon majority vote of the closest “neighbors” in a higher dimensional space [138]. Similar to SVMs, a KNN classifier maps high dimensional datasets to a higher dimensional space where distances between data points are calculated using Euclidean distance or another distance measure. The data points with the shortest distance to the tested sample are defined as neighbors, which are already assigned a class and have defined coordinates in the higher dimensional space through the training phase. Based on the majority class amongst the defined neighbors, the tested data point is assigned to a class. In the context of classifying bacterial Raman spectra, KNN classifiers have shown accuracy comparable to the previously discussed methods. Ciloglu et al. utilized a KNN classifier, along with other methods, to classify samples between strains of methicillin-resistant *Staphylococcus aureus* (MRSA) and methicillin-susceptible *Staphylococcus aureus* (MSSA). Compared to the other classifiers (SVM, decision tree, and Naïve Bayes), the KNN classifier performed best with a 97.8% accuracy [139]. Fu et al. used a combination of principal component analysis (PCA) and the KNN algorithm to classify six species of commonly known urinary tract infection (UTI) bacteria, achieving an accuracy of 85%. An important hyperparameter to consider for the KNN algorithm is k: the number of neighbors used for the majority decision. The main drawback of the KNN approach is a slow prediction phase along with general computational complexity [140]. Overcoming the computational complexity with the KNN algorithm, and with any of the prior algorithms, suggests dimensionality reduction through techniques such as PCA or t-distributed stochastic neighbor embedding (t-SNE). Dimensionality reduction techniques reduce the feature space of a dataset to a smaller number while still explaining the variability in the original data. Reducing the feature space to a smaller number would greatly minimize computational time and resources required for the algorithms. The caveat to this benefit is that, while dimensionality reduction techniques are optimized to explain the variability in the original data, there is a level of information loss which could result in lowered accuracy. As such, the amount to which the feature space is reduced should be determined on a trial-and-error basis.

Clearly, there are multiple possible methodologies within the machine learning space that can be applied to Raman spectra to yield a higher quality of bacterial identification, should suitable data be available. The leading AI implementation remains for artificial neural networks (ANNs). ANNs are algorithms inspired by human neuronal connections, where artificial neurons are arranged into three layer types (input, hidden, and output) to form a weighted graph [141]. Inputs into each artificial neuron are summed and an activation function takes that weighted sum, incorporates a set bias, and determines whether that neuron should send a signal based on thresholds defined by the general activation function [142].

This behavior simulates the all-or-nothing property of neuronal action potentials in human neuronal systems. The firing of particular sets of neurons allows the ANN to classify samples based on final signals emerging from the output layer. ANNs have shown great potential in areas such as cancer diagnosis, histology slide classification, and RNA expression-based classification [143]. ANNs have been modified to increase accuracy and optimize training time: convolutional neural networks (CNNs) and recurrent neural networks (RNNs). RNNs are modified ANNs with recurrent connection within the hidden layer so that the artificial neuron can consider the sequence in which the input is presented [144]. This allows RNNs to sequentially format data such as time-series data. On the other hand, CNNs utilize filters to pass on only the most important information between neuron layers. CNNs are particularly useful in image classification, which have drastically large amounts of input that are easily filtered using the CNN architecture. In the context of classifying bacteria using Raman spectra, CNNs in particular have shown the best performance. Ho et al. utilized a convolutional neural network (CNN) to identify 30 different pathogens, intra- and inter-species, at an accuracy of 89.1% while also achieving 99.7% accuracy when classifying pathogens by antibiotic susceptibility [119]. A summary of the discussed various algorithms (RF, SVM, GBM, KNN, and CNN) used to identify bacteria are shown in Table 2. As noted in this section, the work of integrating microfluidics with Raman spectra and then using artificial intelligence is in a nascent stage and presents significant opportunity for future avenues of discovery.

## 4. Summary and Conclusions

Since the advent of microfluidic devices in the mid-1990s, rapid progress in the field has led to many translational technology advances. In this mini review, we have focused on emerging methods while also discussing both basic science and technological advances. The review of literature emphasizes the use of multi-physics phenomena that govern many demonstrations of technology for bacterial identification.

The continued prevalence of bacterial infection or contamination in environmental or food applications has posed a serious public health concern. Therapeutic remedies struggle to keep up with antimicrobial resistance, and the necessity for early bacterial identification has become more urgent. Historically, conventional methods for bacterial identification have been limited by time-consuming culturing methods and high amounts of bacteria required for accurate determination of the organism and strain. Lab-on-chip methods provide a viable option to combat these drawbacks by enabling small sample sizes to yield accurate detection at lower pathogen concentrations. There are three specific advantages that the microfluidic devices offer in contrast to conventional methods: improved limit of detection (LoD), reduced processing time, and ability to identify non-culturable bacteria.

Furthermore, in this mini review, we provided a review of recent methods combining microfluidic channels with bacterial identification technologies to showcase the multi-physics capabilities that bridging disciplines brings together for several advances. Specifically, we focused on the use of polymerase chain reaction (PCR) and its derivatives, loop-mediated isothermal amplification (LAMP), mass spectrometry, and Raman spectroscopy as emerging methods. Furthermore, we discussed the emerging technique of Raman spectroscopy when used in combination with artificial intelligence (AI) methods. In all these methods, microfluidics enables use of smaller volumes and innovative flow physics to concentrate or prepare bacterial samples for high sensitivity measurements for bacterial identification.

Overall, PCR, LAMP, and MALDI-ToF MS have all demonstrated promise in identifying bacteria without the use of traditional bacterial cultures when coupled with microfluidic devices; however, each method has drawbacks as described throughout. For PCR, limitations arise in the complexity of the technology due to the integration of a complex thermocycler [90,91]. Additionally, previous studies have demonstrated issues due to false positives [85] and diminished discrimination between viable from non-viable bacteria [86]. While LAMP technologies eliminate the necessity of a thermocycler, in most cases, fluorescent dyes are required for pathogen detection, which increases preparation time and can potentially lead to false positive results [145]. Different from both PCR and LAMP, mass spectrometry methods such as MALDI-ToF MS provide pathogen identification without amplifying genetic material. However, MALDI-ToF MS is limited by the large amount of biomass required for an accurate identification [98] and difficulties when incorporating mass spectrometry technology on chip [101], though researchers are developing miniature MS devices for on-chip integration. Therefore, while progress has occurred in the development of culture-free bacterial identification methods, alternate methods continue to be explored. One emerging methodology, Raman spectroscopy, uses optical methods. A summary of the discussed advantages and disadvantages of the PCR, LAMP, MALDI-ToF MS, and Raman spectroscopy is presented in Table 3.

Despite these advances, routine implementation of microfluidic devices for bacterial identification remains limited as currently there are no standards or uniform metrics for the evaluation of device performance. Moreover, the use of microfluidics remains an active area for bacterial research with many opportunities yet to be availed.

## Figures and Tables

**Table 1:** Summary with examples of microfluidic devices using PCR (polymerase chain reaction), LAMP (loop-mediated isothermal amplification), MALDI-ToF MS (matrix-assisted laser deposition/ionization mass spectroscopy), and Raman spectroscopy for bacterial identification.

Identification Technique	Device Details	Target Organism	Limit of Detection (LoD)	Reaction Time	Sample Input and Volume	Reference
PCR	PDMS multiplex microfluidic PCR chip	*Salmonella*	100 CFU/mL	~47 min	Extracted DNA–84 μL	[34]
	Integrated PDMS microfluidic chip with membrane-based filteration module, bacterial-capture module utilizing a micro-mixer with FcMBL-coated magnetic beads, and multiplex PCR module	*Escherichia coli*, *Klebsiella pneumoniae*, *Pseudomonas aeruginosa*, *Staphylococcus epidermidis*, *Staphylococcus saprophyticus*	1–5 CFU/mL	4 h	Bacteria inoculated human blood samples–5.4 mL	[35]
	Coiled PTFE capillary tube with multiplex segmented continuous-flow PCR	*Salmonella enterica*, *Listeria monocytogenes*, *Escherichia coli* O157:H7, *Staphylococcus aureus*	100 gene copies/mL	~19 min	Extracted DNA from artifically contaminated food samples–25 μL	[36]
	Integrated PDMS microfluidic chip with 12 singleplex reaction chambers	*Mycobacterium tuberculosis*	100 CFU	90 min	Bacteria inoculated ddH_2_O, 1xPBS, normal saline (0.9%NaCl), sputum, and whole blood–20 μL	[37]
LAMP	Continuous liquid interface production (CLIP) -based AM PTFE capillary cartridge with integrated LAMP	*Escherichia coli*	50 CFU/mL	40–50 min	Bacteria inoculated whole blood samples–8 μL	[38]
	PDMS and capillary channel-based microfluidic chip with singleplex LAMP integration	*Escherichia coli malB*	1 pg/mL	~60 min	Extracted DNA–60 nL	[39]
	PMMA spiral microchannel with 24 multiplex LAMP reaction chambers	*Escherichia coli* O157:H7, *Salmonella typhimurium*, *Vibrio parahaemolyticus*	500 gene copies/reaction	~60 min	Extracted DNA–75 mL	[40]
	Cyclo olefin polymer (COP)-based chip containing a straight microchannel connected by 15 multiplex LAMP reaction wells	*Salmonella*, *Campylobacter jejuni*, *Shigella*, *Vibrio cholerae*	10–100 genomes/mL	~20 min	Extracted DNA–15 μL	[41]
	PDMS channel for sample delivery to mutiplex LAMP reaction chambers	*Escherichia coli*, *Proteus hauseri*, *Vibrio parahaemolyticus*, *Salmonella* subsp. *Enterica*	~3 copies/mL	~120 min	Bacteria inoculated solution–600 nL	[42]
MALDI-ToF MS	Repetitive PDMS herringbone channel containing vancomycin modified magnetic beads with off-chip MALDI-ToF MS	*Staphylococcus aureus*, *Staphylococcus hominis*, *Staphylococcus epidermidis*, *Enterococcus gallinarum*	10^4^–10^5^ CFU/mL	90 min	Bacteria inoculated solution–250 μL	[43]
	Microchannel silicon nanowire (McSiNW) microfluidic chip with off-chip MALDI-ToF MS	*Escherichia coli* (cultured and uncultured)	10^3^–10^6^ CFU/mL	60 min	Bacteria inoculated urine–500 μL	[44]
Raman Spectroscopy	PDMS microfluidic microwell device with bonded SERS substrate	*Escherichia coli*	10^8^ CFU/mL	3.5 hrs	Bacteria inoculated solution–5 mL	[45]
	Membrane filtration-based PMMA microfluidic with SERS-active substrate	*Escherichia coli*, *Staphylococcus aureus*	10^3^ CFU/mL	30 min	Bacteria inoculated solution–10 mL	[46]
	PDMS microchannel with SERS functionalized components	*Listeria monocytogenes*, *Listeria innocua*	10^5^ CFU/mL	30 min	Combined mixture of bacteria and SERS-tagged gold nanostars	[47]

**Table 2:** List of aforementioned AI algorithms showing applications to bacterial classification via Raman spectroscopy along with corresponding methodology and considerations for each algorithm.

AI Algorithm	Target Organism	Accuracy	Methodology	Considerations	Reference
Support Vector Machince (SVM)	*Escherichia coli*	81.1%	Use hyperplane optimization to demarcate between class data	Not inherently designed for multi-class (2+) classification	[126,128]
Random Forests (RFs)	3 bacterial and 3 archaeal species	98.9%	Average of multiple decision trees trained on random subsets of training data	Lack of interpretability and tendency to overfit model	[133,134]
k-nearest-neighbors (KNN)	10 methicillin-resistant *S. aureus*, 6 methicillin-sensitive *S. aureus*, and 6 *L. pneumophila* isolates	97.8%	Maps high dimensional data to a higher dimensional space and define class members based on proximity by a distance measure	Optimization of *k* along with computational complexity requires extended effort	[139,140]
Gradient Boosted Machines (GBM)	15 strains of *Klebsiella pneumoniae* based on Carbapenem resistance	99.40%	Apply loss function to a base learner (decision tree, regression model, etc.) and repeat training until loss function reaches minima	Computational complexity due to number of iterations needed to minimize loss function	[137]
Convolutional Neural Networks (CNN)	30 species and strains of various bacteria	89.1%	Model neuronal connections based on activation function for input classification	Complex theory behind neural networks requires expert knowledge before use	[119]

**Table 3:** Summary of advantages and disadvantages relating to discussed microfluidic methods using PCR (polymerase chain reaction), LAMP (loop-mediated isothermal amplification), MALDI-ToF MS (matrix-assisted laser deposition/ionization mass spectroscopy), and Raman spectroscopy for bacterial identification.

Identification Technique	Advantages	Disadvantages
PCR	Small amount of biomass required for amplification and detection [73], Portability enables rapid PoC testing [74,75]	Complex fabrication due to thermocycler utilization [90,91], prior sample preparation often required, potential for false positive results [85], inefficient discrimination between viable and nonviable cells [84]
LAMP	Small amount of biomass required for amplification and detection, high operating speed, eliminates the necessity for a thermocycler [90,91]	Difficult thermoregulation [88,89], prior sample preparation often required [145], inaccurate fluorescent dye detection creates potential for false positive results [145]
MALDI-ToF MS	Does not require amplification of genetic material [6], high specificity for identification [98]	Large amount of biomass required for detection [98], Difficult to integrate on chip [101]
Raman Spectroscopy	Non-destructive to samples, real-time acquisition without need for extensive sample manipulation, acquisition in confined spaces	Low efficiency of Raman scattering makes measurements harder [109], application hindered by variable bacterial growth conditions [116], causing metabolomic changes in bacteria that can result in variations in spectral reading

## Data Availability

Data sharing not applicable.
